# Improved intraocular bioavailability of ganciclovir by mucoadhesive polymer based ocular microspheres: development and simulation process in *Wistar* rats

**DOI:** 10.1186/s40199-015-0132-7

**Published:** 2015-10-24

**Authors:** Usha Ganganahalli Kapanigowda, Sree Harsha Nagaraja, Balakeshwa Ramaiah, Prakash Rao Boggarapu

**Affiliations:** Department of Pharmaceutical Technology, Karnataka College of Pharmacy, #33/2, Tirumenahalli, Hegde Nagar Main Road, Bengaluru, 560064, Karnataka India; Department of Pharmaceutical Sciences, College of Clinical Pharmacy, King Faisal University, Al-Ahsa, 31982 Saudi Arabia; Department of Pharmaceutics, Karnataka College of Pharmacy, #33/2, Tirumenahalli, Hegde Nagar Main Road, Bengaluru, 560064, Karnataka India

**Keywords:** Franz cells, Superimposition, Release kinetics, Ocular pharmacokinetic, Simulation

## Abstract

**Background:**

The poor ocular bioavailability of the conventional eye drops is due to lack of corneal permeability, nasolacrimal drainage and metabolic degradation. To overcome this issue, drug encapsulated in mucoadhesive polymer based ocular microspheres have the advantages of improved drug stability, easy administration in liquid form, diffuse rapidly and better ocular tissue internalization.

**Methods:**

The ganciclovir chitosan microspheres (GCM) were prepared by modified water-in-oil emulsification method. The formulation was optimized and characterized by investigating *in vitro* release study, release kinetics, XRD and microspheres stability. Ocular irritancy, *in vivo* ocular pharmacokinetic parameters and histopathology study was evaluated in *Wistar* rats. The use of pharmacokinetic/pharmacodynamic indices and simulation process was carried out to further ensure clinical applicability of the formulation.

**Results:**

The *in vitro* release study showed initial burst (nearly 50 %) in first few minutes and followed Fickian (R^2^ = 0.9234, n-value = 0.2329) type of diffusion release mechanism. The XRD and stability studies showed favorable results. The *Wistar* rat eyes treated with GCM showed significant increase in ganciclovir AUC (~4.99-fold) and C_max_ (2.69-fold) in aqueous humor compared to ganciclovir solution and delay in T_max_. The C_max_/MIC_90_, AUC_0–24_/MIC_90_, AUC above MIC_90_ and T above MIC_90_ were significantly higher in GCM group. The aqueous humor concentration-time profile of ganciclovir in GCM and ganciclovir solution was simulated with every 28.1 and 12.8 h, respectively. The simulated concentration-time profile shows that in duration of 75 h, the ganciclovir solution require six ocular instillations compared to three ocular instillations of the GCM formulation. The photomicrograph of GCM and ganciclovir solution treated rat retina showed normal organization and cytoarchitecture.

**Conclusions:**

Correlating with *in vitro* data, the formulation showed sustained drug release along with improved intraocular bioavailability of ganciclovir in *Wistar* rats.

## Background

Herpes simplex keratitis and cytomegalovirus retinitis have been the most common viral infections observed worldwide [1]. Recurrent and relapse of ocular viral infections can lead to corneal perforation resulting in blindness. The poor bioavailability of the conventional eye drops is due to lack of corneal permeability, nasolacrimal drainage and metabolic degradation. Hence, an optimum treatment must be considered for effective management of ocular viral diseases. Ganciclovir, a broad spectrum antiviral drug was considered to be highly active against *cytomegalovirus* and *herpes simplex virus*. Ganciclovir, an acyloguanosine derivative after *in vivo* administration gets modified into ganciclovir triphosphate. It competitively inhibits the virus deoxyribonucleic acid (DNA) polymerase by impairing the viral DNA synthesis [2].

Ganciclovir requires frequent oral administration, has it shows very poor bioavailability (6–9 %) [3]. Administration of drug via oral, intravenous or extravascular injection leads to low drug concentration at the site of infected eye [4]. This finding was supported by a study conducted by Young et al. [5], reports that the contralateral retinitis was higher in patients treated with intravenous maintenance therapy of ganciclovir (15–68 %) compared to intravitreous ganciclovir (11 %). Moreover, due to its short half-life, frequent intravitreal injections leads to risk of retinal detachments, hemorrhages, or endophthalmitis. Ganciclovir encapsulated with PLGA microspheres for ocular delivery has been investigated [6]. Reported clinical studies [2, 7, 8] found that the ganciclovir efficacy can be accomplished by formulating the drug as an ophthalmic topical preparation. Hence, a sustained intraocular drug concentration can be achieved by using a desired polymer in the form of microspheres for ocular instillation, which also seems to reduce the ocular toxicities. Ganciclovir combined with chitosan showed two fold increased oral bioavailability [9].

The inherent biological activity of chitosan [poly (β-(1 → 4)-2-amino-2-deoxy-D-glucose)] signifies its role in ocular therapeutics. With various degrees of N-acetylation of glucosamine residues, it is considered as a linear binary heteroploysaccharide composed of β-1,4-linked glucosamine. Chitosan being a promising natural biodegradable polymer with hydrophilic in nature improves stability, precorneal retention and enhances interaction with eye mucosa. Moreover, the sustained release, mucoadhesive, in situ gelling, transfection and permeation enhancing properties of chitosan are recognized as few parameters of the polymer suitable for ocular drug delivery. The unique physical properties of chitosan bring transitions in the paracellular and transcellular pathway without disturbing cellular integrity. This innate chitosan property allows the drug to be transported to the inner eye and helps the drug to get accumulated at corneal epithelia [10, 11].

Chitosan has the ability to augment intraocular drug penetration by binding with cornea and reversibly loosening the tight corneal conjunctions. Additionally, non toxic, low eye irritation and ability to release the drug at a sustainable fashion qualifies it as one of the ideal polymer for ophthalmic preparation [12, 13]. Chitosan has been used in many ophthalmic preparations such as indomethacin nanoemulsions [14]; indomethacin nanocapsules [15]; cyclosporine A nanoparticles [16]; ofloxacin microspheres [17] and acyclovir microspheres [18]. Zirgan™ and Virgan® are commercially available ganciclovir ophthalmic gels. Zirgan™ has been approved in European countries since 1995 and in 2009 it was approved in United States [1]. Additionally, the ganciclovir implant (Vitrasert) received USFDA approval for the treatment of cytomegalovirus retinitis in immunodeficiency patients.

Intraocular or periocular injections of microparticles or nanoparticles can lead to vitreous clouding and foreign body response. Due to low biodistribution coefficient, the topical administration of ganciclovir has limitations. Frequent administrations of the conventional eye drops are required due to their short retention time and decreased ocular drug bioavailability [19]. Thus, the microspheres are preferred delivery system for ocular drug delivery. The polymeric microspheres have the advantages of easy administration in liquid form, diffuse rapidly and better ocular tissue internalization. The entrapped drug in the form of monolithic-type or reservoir type in the microspheres can act as depot and sustain the release of drug. Hence, literatures [2, 3] support the use of ganciclovir as microsphere formulation for improved antiviral effectiveness.

The requirement of ganciclovir ocular preparation for topical application with better therapeutic efficacy and good safety profile was evident. Thus, this study was an attempt to investigate formulation of ganciclovir using mucoadhesive polymer intended for sustained and improved intraocular delivery. The preparation was characterized by *in vitro* drug release, release kinetics, X-ray diffraction (XRD) and stability study. Further, ocular irritancy, *in vivo* ocular pharmacokinetic, histopathology along with pharmacokinetic/pharmacodynamic indices and simulation process was utilized to identify the efficacy and tolerability of the optimized formulation.

## Materials and methods

### Materials

Ganciclovir was acquired as a gift sample from Dr. Reddys Laboratories, Hyderabad, India. Chitosan (93 % deactylation) was purchased from Yarrow Chem Products, Mumbai, India. Other reagents used were of analytical grade.

### Analytical method

Reverse-phase high performance liquid chromatography (RP-HPLC) was used for quantitative analysis of ganciclovir [20]. The C8 column (15 cm × 4.6 mm; 5 μ) was utilized for the analysis. The mobile phase consisted of mixture of 0.1 *M* sodium dihydrogen phosphate monohydrate and 0.04 *M* triethylamine in the ratio of 50:50 (pH maintained at 6.6). The column temperature was retained at 40 °C. The injection volume was 20 μL and the flow rate was maintained at 1 mL/min. The sample was detected using UV at 254 nm. The acyclovir was considered as an internal standard. The standard calibration curve was linear in the concentration range of 50 to 1000 ng/mL.

### Experimental design

The central composite design was used to optimize ganciclovir loaded chitosan microspheres (GCM) by altering variable factors and their effect on encapsulation efficiency and 12^th^ hour *in vitro* drug release. The model contained eight factorial points, six axial points and six centre points with total 20 experiments. The mean value was set as 0 and, +1 and −1 was considered as higher and lower levels for each factor respectively. The selected factors with their levels along with optimized levels are summarized in Tables 1 and 2.

**Table 1:** Variable factors with their levels used for optimization of GCM

Variable factors	Level					Optimized level
	−1.41	−1	0	1	1.41	
Chitosan concentration (mg)	79.55	250	500	750	920.45	750
Stirring speed (rpm)	318	1000	2000	3000	3682	3000
Span 80 volume (mL)	0.20	0.40	0.70	1.0	1.20	1.00

**Table 2:** Response factors with expected and observed values for optimized GCM

Response factors	Expected value	Observed value	Residual value
Encapsulation efficiency (%)	81.4	80.00	−1.4
12^th^ hour *in vitro* drug release (%)	77.27	78.00	0.73

### Preparation of optimized GCM

The optimized GCMs intended for ocular sustained release were prepared by modified water-in-oil emulsification method [21]. Seven hundred and fifty milligrams of 93 % deacetylated chitosan was dissolved in 50 mL of 1 % w/v acetic acid maintained at pH 2.72. Five hundred milligrams of ganciclovir was added to the above solution with agitation and the mixture was sonicated for 10 min. The resultant mixture was centrifuged (1000 rpm, 10 min) to separate any remains of undissolved chitosan. The oil phase consisted of 20 mL of dichloromethane and 20 mL of liquid paraffin with 1 mL of 1 % v/v of Span 80. The aqueous phase was introduced slowly as drop wise in to the oil phase under continuous homogenization at 3000 rpm. Further, the water in oil emulsion was homogenized to crosslink the microspheres with 5 % v/v of glutaraldehyde. The emulsion was again added to 20 mL of pre-heated liquid paraffin at 170 °C with stirring to remove dichloromethane and aqueous solvent. The formed microspheres were filtered through 0.45 μm Millipore filters. To remove residual liquid paraffin, the microspheres was then washed 5–6 times with 100 mL of diethyl ether and was vacuum dried for 24 h.

### *In vitro* drug release of the optimized GCM

Using Franz diffusion cells, the *in vitro* release of ganciclovir from the microspheres was investigated. The molecular weight cut-off of the dialysis membrane was between 12,000–14,000 Da (Himedia Laborateries Pvt. Ltd, Mumbai, India). The dialysis membrane acted as a barrier to separate the donor and acceptor compartment. The simulated tear fluid (STF) was used as the dissolution medium, which was prepared by adding 0.67 % of NaCl; 0.2 % of NaHCO3; 0.008 % of CaCl_2_. 2H_2_O and the resultant solution pH was adjusted to 7.4. A weighed amount of microspheres dispersed in 1 mL of the STF was kept in the donor compartment. The STF (100 mL) was filled in the acceptor compartment and stirred magnetically at 100 rpm maintaining the temperature at 37 ± 0.5 °C [22]. For a period of 12 h, 1 mL of the sample was withdrawn every hour from the acceptor compartment and subjected to UV spectroscopy scanned at 254 nm. The same amount of the fresh STF was replaced into the acceptor compartment.

### Release kinetics of the optimized GCM

Various models such as first order model (1); Higuchi square root model (2); Baker and Lonsdale (3); Koresmeyer-Peppas (4) and; Hixon and Crowell cube root model (5) models were used to study drug release mechanism from the microspheres. The data was fitted to these models and was analyzed using sigma plot.

1 $$ {Q}_t = {Q}_0\ {\mathrm{e}}^{-{k}_1t} $$

2 $$ {Q}_t = {k}_H\sqrt{t} $$

3 $$ \frac{Q_t}{Q_{\infty }}=1 - \frac{6}{\pi^2} exp\left(\frac{-{\pi}^2 \times Dt}{r^2}\right) $$

4 $$ {Q}_t = {Q}_0 + a{\left(\frac{t}{r^2}\right)}^n + b{\left(\frac{t}{r^2}\right)}^{2n} $$

5 $$ \sqrt[3]{Q_0} - \sqrt[3]{Q_t}={\mathrm{k}}_{\mathrm{HC}}t $$

*Q*_*t*_ is the total amount of drug released after time (%); *Q*_0_ the initial amount of drug (%); *k*_1_ the first order release rate constant (h^−1^); k_H_ the rate constant obtained according to the Higuchi equation (%h^−1/2^); *Q*_∞_ is the percent release at infinite time; *D* is the diffusion coefficient in the polymer in cm^2^/*s; r* is the radius of the sphere in cm; *n* and *2n* are the release exponent for Fickian diffusion and case II transport, respectively; *a* and *b* are constants related to the drug and the structural and geometric properties of the microparticles; and k_HC_ is the rate constant obtained according to the Hixon and Crowell equation (%h^−1^) [23–25].

### XRD

XRD of ganciclovir powder, chitosan and the optimized GCM was performed (Philips XPert Pro, Netherlands) at 40 kV voltage with 30 mA of the current, utilizing a nickel-filtered CuKα radiation. With 0.02° interval, the sample was scanned over a 2θ range of 10–80° at a rate of 2°/min.

### Stability study of the optimized GCM

The stability study was conducted as per ICH Q1AR guideline, intended to test the stability for new substances and product. The optimized preparation was stored at 25 ± 2 °C and 60 ± 5 % RH for twelve months and at 5 ± 3 °C for a period of six months. The required volume of microsphere dispersion was stored in closed glass bottles and sealed tightly. At regular intervals, the sample was subjected for determination of encapsulation efficiency, mean particle size distribution and for any physical changes. The test was carried at three month intervals for a period of 12 months for long term storage condition at room temperature and at 0, 2, 4 and 6 months for accelerated condition at refrigeration storage.

### *In vivo* ocular pharmacokinetic studies of the optimized GCM

Prior to the study, the ethical clearance for *in vivo* experimental protocol was obtained from Institutional Animal Ethics Committee (IAEC) which is registered under CPCSEA, India. The *Wistar* rats (male and female) free from ocular defects, 11–13 weeks older and weighing around 180–200 g was utilized for the study.

### Ocular irritation

The microspheres ocular tolerability [26] was evaluated by identifying the ocular irritancy. The 1 % w/v of the sample was prepared by dispersing the microspheres in isotonic normal saline (ganciclovir solution) and was immediately used for the study. The GCM sample (25 μL) was directly instilled into right eye of the rat and for uniform dispersion on cornea; the rats were forced to wink once. The left eye was instilled with normal saline alone and acted as a control. Post instillation, both the eyes were observed for frequency of winking in 5 min.

### *In vivo* evaluation

A total of 32 *Wistar* rats were housed in standard cages and had free access to food and water. The rats were allowed for free head and eye movement. To carry out *in vivo* study [27], 75 μL (3 × 25 μL drops at 90 s intervals) of the GCM (1 % w/v) was freshly prepared and was immediately instilled with micropipette into lower conjunctival sac of the right eye without touching the eye. The 1 % w/v of the ganciclovir solution served as a control and as above mentioned quantity and procedure was instilled to the left eye. At 0.5 h, 1 h, 1.5 h, 2 h, 3 h, 4 h, 5 h, 6 h, 12 h, 24 h post ocular instillation, the animal was sacrificed by cervical dislocation and the entire eyes were removed. The aqueous humor from the isolated eyes was separated and was stored in micro centrifuge tubes at –20 °C until further analysis. After the process of extraction and isolation, the sample was subjected to the RP-HPLC analysis.

### Extraction and isolation

The *in vivo* pharmacokinetic estimation of the ganciclovir in aqueous humor was performed as mentioned above by RP-HPLC method. The extracted aqueous humor (100 μL) was added to 100 μL of 50 % trichloroacetic acid, shaken well and was centrifuged at 2000 g for 10 min to deproteinize the sample. The supernatant was neutralized with 50 μL of 2 *M* sodium hydroxide and vortexed. Later, the sample was extracted with 5 mL of chloroform and centrifuged at 3000 g for 5 min. The extracted sample was mixed with 10 μL of the mobile phase. Finally 20 μL of the mixture and 20 μL of the acyclovir as an internal standard were injected into HPLC system [20].

### Pharmacokinetic and Statistical analysis

The pharmacokinetic parameters were calculated using one compartment open model. The ganciclovir, area under the curve (AUC) in aqueous humor was determined from the beginning of the drop instillation (t_0_) to the last observation (t_last_) by linear trapezoidal rule with extrapolation to infinite time. Additionally, ganciclovir half life (t_1/2_), relative bioavailability, the maximum peak concentration (C_max_) and time to achieve maximum peak concentration (T_max_) was also calculated. The terminal rate constant (K_e_) and apparent absorption rate (K_a_) of ganciclovir from aqueous humor was estimated from the terminal portions of the respective log (aqueous humor concentration) vs. time linear regression plots [28].

The Kinetica 5.0 PK/PD analysis software was also utilized for the calculation of pharmacokinetic parameters. The estimated pharmacokinetic/pharmacodynamic (PK/PD) indices such as C_max_/MIC_90_, AUC_0–24_/MIC_90_, AUC above MIC_90_ and T above MIC_90_ was calculated to determine the *in vivo* efficacy of the GCM. The principle of superimposition using Microsoft excel software was used to evaluate the simulation of aqueous humor concentration- time profile at different dosing interval [29]. Based on the time where the ganciclovir aqueous humor concentration was maintained twice the MIC_90_ (1.22 μg/mL), the subsequent dose was calculated. Student’s *t* test (*p* < 0.05) was considered for statistical significance.

### Histopathology

The isolated eyes were stored in 10 % formalin and were subjected to histopathological examination. The retina was isolated, dyed with hematoxylin-eosin and was observed under light microscopy with 200× magnification for cytoarchitecture changes.

## Results and discussion

### Preparation of the optimized GCM

The modified water-in-oil emulsification method was found to be suitable and simple technique for encapsulating ganciclovir using chitosan. The degree of chitosan deacetylation and molecular weight are considered to be two fundamental parameters that influence the properties and functionality of chitosan. These parameters along with crystallinity influence chitosan degradation and ocular epithelial cell permeability. More than 60 % of deacetylation of chitosan is considered to be ideal for ocular delivery as decrease in deacetylation leads to decreased water solubility of the polymer. Interestingly, trimethylated chitosan with PEGlation a 3.4-fold increase in its mucoadhesive property was observed, but no such pronounced observation was found in respect to its permeation enhancing property [11]. However, this study used 93 % deacetylated chitosan for the purpose of encapsulation of ganciclovir. On ocular instillation, the liquid form of chitosan transforms into gel form at physiological pH of 7.4 and significantly helps in longer residence time and biodistribution of the drug on corneal surface. The negatively charged cornea and sclera interacts with positive charged amino groups of the chitosan, hence enhances the ocular bioavailability [10, 30]. A study by Mathew et al. [31], showed the increased sustained drug release by using optimum glutaraldehyde as cross linking agent. Earlier many studies [32, 33] have successfully prepared chitosan microspheres from emulsion cross-linking method.

### *In vitro* release study of the optimized GCM

**Fig. 1:**
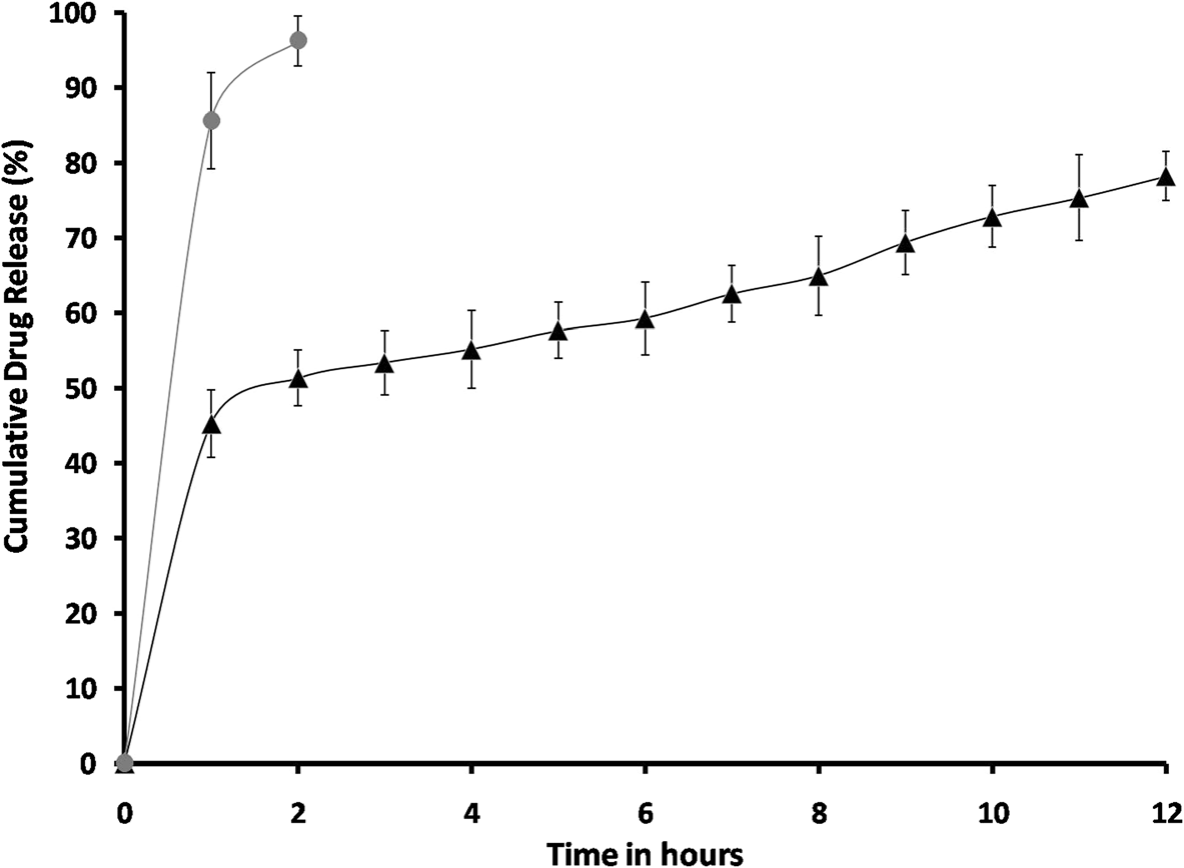
Cumulative amount of drug released ( ) GCM and ( ) ganciclovir solution (bars represent mean ± SD; *n* = 3)

*In vitro* drug release of the optimized GCM and ganciclovir solution using STF was investigated separately (Fig. 1). The *in vitro* data showed biphasic pattern of ganciclovir release from GCM. The initial drug loading followed by marked prolongation of drug residence time was achieved by an initial immediate burst effect (nearly 50 %) in few minutes and then slower release over few hours (up to 90 %). The appropriate physicochemical properties of the microspheres help in achieving adequate drug bioavailability and biocompatibility with ocular mucosa. The initial burst release was beneficial in attaining required therapeutic concentration of the drug in negligible time. The rapid and instantaneous initial release was due to the modified water-in-oil emulsification preparation method that resulted in deposition of drug on surface of the microspheres. The drug adhered to the surface of the microspheres are primarily released into aqueous media by desorption and diffusion causing the initial burst [29, 34]. The decrease in particle size enhances this effect as the formation of large surface area. Similar finding has been observed for different drugs encapsulated in chitosan microspheres [35]. The sustained action in the later stage was due to diffusion of ganciclovir from the polymeric matrix and biodegradation of chitosan. Genta et al., [18] has demonstrated the mucoadhesive and sustain release activity of the chitosan.

### Curve fitting analysis of the optimized GCM

**Fig. 2:**
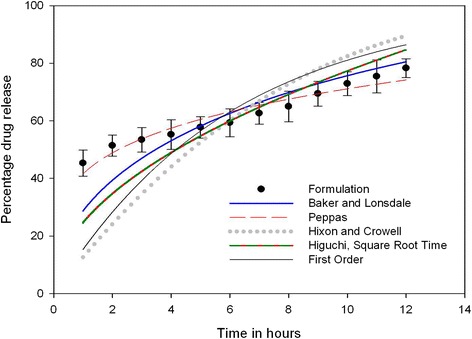
*In vitro* release profile of the optimized GCM-curve fitting models (bars represent mean ± SD; *n* = 3)

The Fig. 2 shows curve fitting of the optimized formulation *in vitro* drug release kinetics. Among the models, Koresmeyer–Peppas model was best fitted by significant regression coefficient (R^2^ = 0.9234). Using Fick’s law, Koresmeyer-Peppas model helps in investigating drug release mechanism from the polymeric system in the first 10h of the *in vitro* study. The n-value (0.2329, *p* < 0.0001) of the optimized formulation was less than 0.45, indicating Fickian type of diffusion mechanism of drug release. The Koresmeyer-Peppas model explains when the drug release mechanism is a combination of drug diffusion - Fickian transport-, and in Case II transport - non-Fickian-, controlled by the relaxation of polymer chain. Chitosan polymeric matrix usually represents diffusion and erosion type of drug release [36]. The sustained action of the ocular delivery also depends on the surface characteristics of the microspheres. The positive zeta potential can facilitate an effective adhesion to the cornea surface and also could improve some limitations related to ocular administration, such as prevent tear washout (due to tear dynamics). Subsequently, the positive charge interact with the cell membrane leading in a structural reorganization of tight junction-associated proteins helps in permeation of the drug through corneal surface and improves intraocular drug bioavailability [11, 37].

### Stability study of the optimized GCM

**Fig. 3:**
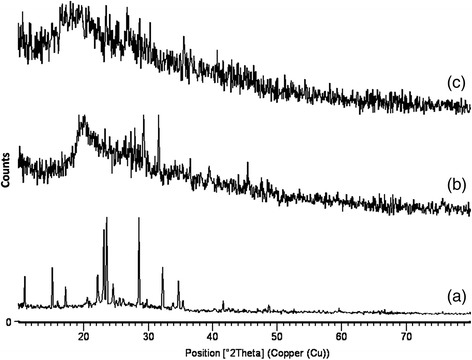
XRD of (**a**) ganciclovir; (**b**) chitosan; (**c**) optimized GCM

The stability test observations of the optimized GCM at room temperature and refrigeration conditions are depicted in Tables 3 and 4. On storage, no major deviations were observed in the macroscopic characteristics. A slight increase in mean particle size was noted at 25 °C. The XRD spectral characteristics are shown in Fig. 3. On storage, the extent of microsphere sedimentation was not prominent, on manual agitation they were redispersed easily. XRD spectral characteristic of the ganciclovir pure drug shows many diffraction peaks, indicating the crystallinity of the drug. In contrast, the diffraction peaks were significantly reduced in GCM. XRD of chitosan shows few peaks, which indicates non crystallinity. The GCM formulation showed decreased crystallinity of ganciclovir, which was similar to that of chitosan indicating the incorporation of ganciclovir in the polymer. The increase in mean particle size could be due to increased kinetic energy of system contributing to higher rate of particle collision [26]. Thus, the optimized formula proved to be stable on long term and accelerated storage conditions as well.

**Table 3:** Stability test observations of the optimized GCM at room temperature

Storage	Encapsulation efficiency (%)					Mean Particle size (μm)					Physical change				
	Months					Months					Months				
25 ± 2 °C	0	3	6	9	12	0	3	6	9	12	0	3	6	9	12
	80.79	79.92	79.15	78.57	77.39	20.28	20.15	20.20	20.35	20.40	--	--	--	--	--

--: No physical change

**Table 4:** Stability test observations of the optimized GCM at refrigeration conditions

Storage	Encapsulation efficiency (%)				Mean particle size (μm)				Physical change			
	Months				Months				Months			
5 ± 3 °C	0	2	4	6	0	2	4	6	0	2	4	6
	80.79	80.62	79.89	79.00	20.28	20.19	20.24	20.30	--	--	--	--

--: No physical change

### *In vivo* ocular pharmacokinetic studies of the optimized GCM

The ocular irritation test showed that the GCM sample (12.0 ± 1.0 *vs ganciclovir solution 10.0 ± 1.0*) was well tolerated. The ocular pharmacokinetic of the optimized GCM (1 % w/v) was compared with the ganciclovir solution (1 % w/v) in *Wistar* rats. The dose volume and strength of both the samples were same. The 1 % w/v strength of both the samples would provide optimum C_max_ so as to decrease the nasolacrimal removal of ganciclovir. Subsequently, the relative hydrophilicity of the ganciclovir limits its corneal penetration. The paracellular diffusion of ganciclovir between the tight junctions of the corneal epithelial cells posses a greater challenge [1]. Peyman and Ganiban, [38] showed that the ganciclovir dose upto 400 μg/0.2 mL was found to be non toxic to the retina. Hence, to maintain higher concentration of ganciclovir, 1 % w/v of the GCM was used in this study for *in vivo* evaluation. Young et al., [5] study confirms that the injection of greater than 10 mg of ganciclovir into vitreous humor may result in retinal damage. Throughout this study, the ganciclovir concentration in aqueous humor was below the reported toxic ganciclovir concentration in eye. The 0.15 % ophthalmic gel has shown the mean ganciclovir concentration in tears ranging from 0.92 to 6.86 μg mL^−1^ without any ocular discomfort [1].

**Fig. 4:**
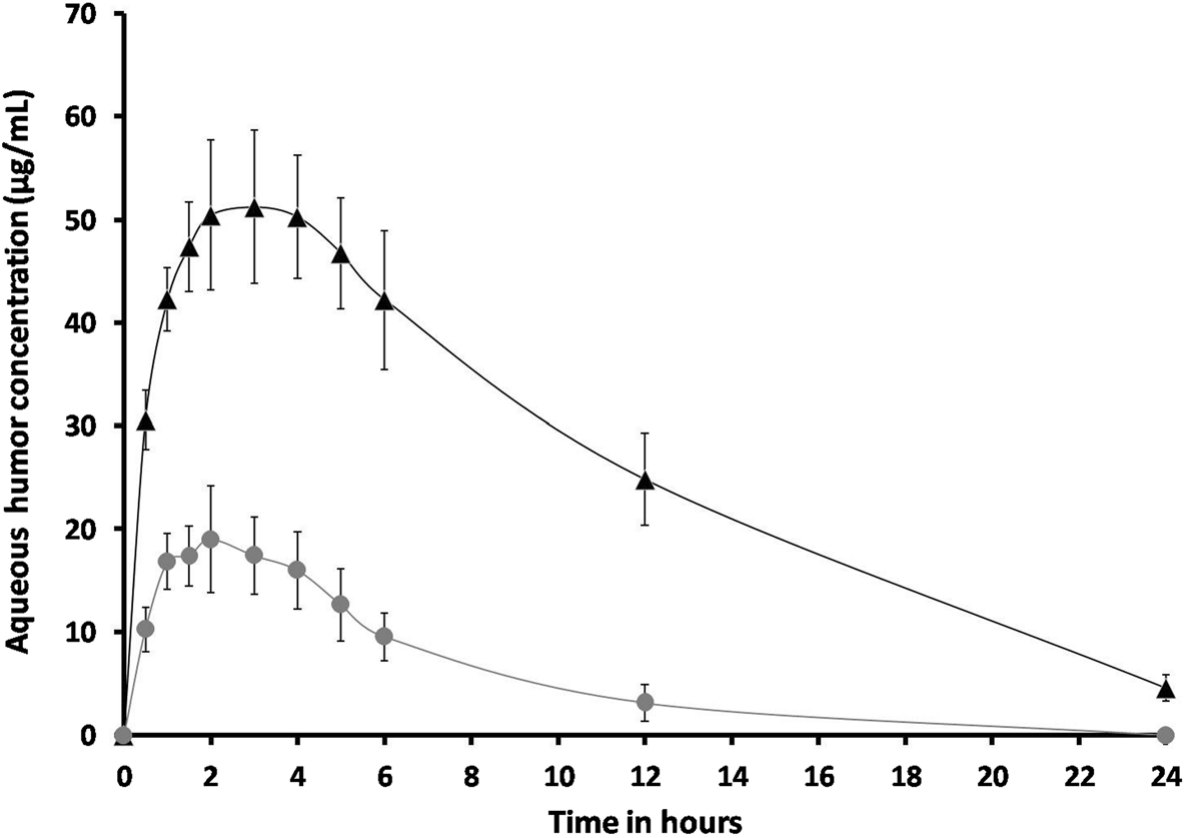
Aqueous humour concentration of ganciclovir after instillation of 1 % w/v of ( ) GCM and ( ) ganciclovir solution (bars represent mean ± SD; *n* = 3)

The aqueous humor concentrations of ganciclovir after instillation of 1 % w/v of GCM and ganciclovir solution were shown in Fig. 4. The Table 5 illustrates the aqueous humor pharmacokinetic parameters. In comparison with ganciclovir solution, the GCM showed significant increase in AUC (~4.99-fold). The absorption rate constant (K_a_) data showed that the GCM trans-corneal permeability was enhanced and was statistically significant than the ganciclovir solution. Subsequently, the terminal rate constant (K_e_) and t_1/2_ did not alter much. The C_max_ of GCM was 2.69-fold of the ganciclovir group (*p* < 0.0001) and the delay in T_max_ infers the sustained release of the GCM. The incorporation of ganciclovir into microspheres significantly increased relative bioavailability.

**Table 5:** Aqueous humor pharmacokinetics parameters after ocular instillation of GCM (1 % w/v) and ganciclovir solution (1 % w/v) in *Wistar* rat

Parameters	Units	GCM	Ganciclovir solution	*P* value
K_a_	h^−1^	0.7252	1.2981	0.0021
K_e_	h^−1^	0.1233	0.1662	0.6270
T_max_	h	3.0	2.0	----
C_max_	μgmL^−1^	51.23	18.98	<0.0001
Relative bioavailability^a^	unitless	4.991	1.000	----
t_1/2_	h	5.7654	3.5426	0.0636
AUC_0-24_	hμgmL^−1^	607.187	121.634	<0.0001
AUC_0-∞_	hμgmL^−1^	645.116	137.692	<0.0001

^a^Relative bioavailability = (AUC_GCM_ × Dose_ganciclovir solution_)/(AUC_ganciclovir solution_ × Dose_GCM_)

This finding signifies the enhanced binding force of the positively charged GCM to the eye surface. Usually, a mucus film as a thin fluid layer covers the surface of the cornea and conjunctiva. The mucin (high molecular mass glycoprotein) being a primary constituent of mucus carries negative charge at physiological pH. Hence, the positively charged chitosan interact with sialic groups and sulfonic acid substructures of mucin and act as an adhesive force to the eye surface. Upon dissolution, the protonation of amino groups (-NH_2_) of the glucosamine to –NH_3_^+^, and the cationic polyelectrolyte readily forms electrostatic interactions with other anionic groups. Thus, the formation of hydrogen bond to the eye surface which is considerably influenced by cationic free amine and hydroxyl groups of chitosan [11, 28, 30, 39].

The estimated pharmacokinetic/pharmacodynamic (PK/PD) indices after ocular instillation of GCM and ganciclovir solution in *Wistar* rat are shown in Table 6. The PK/PD indices play an important role in treatment selection and dosage regimen of ganciclovir as its antiviral activity is concentration dependent. The minimum inhibitory concentration (MIC) solely fails to explain the *in vivo* activity of an antimicrobial agent. In this study, the C_max_/MIC_90_ and AUC_0–24_/MIC_90_ of GCM were maintained higher than the ganciclovir solution. The C_max_/MIC, AUC/MIC, AUC above MIC and T above MIC were higher in the GCM compared to ganciclovir solution and thus indicates the clinical effectiveness. Moreover, for effective antimicrobial activity, the C_max_/MIC_90_ and AUC_0–24_/MIC_90_ of GCM should be higher than 10 and 125, respectively which were complied with GCM formulation [29]. The simulated values also suggest the ideal dosing frequency.

**Table 6:** The estimated pharmacokinetic/pharmacodynamic (PK/PD) indices after ocular instillation of GCM (1 % w/v) and ganciclovir solution (1 % w/v) in *Wistar* rat

PK/PD indices	Units	GCM	Ganciclovir solution
C_max_/MIC_90_	unitless	41.991	15.560
AUC_0−24_/MIC_90_	h	497.694	99.700
AUC_0−24_ above MIC_90_	hμgmL^−1^	573.518	106.239
*T* above MIC_90_	h	28.1	12.8

**Fig. 5:**
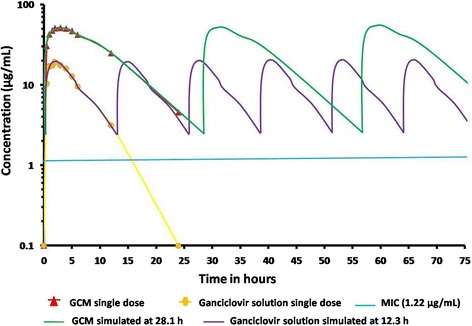
Simulated ocular concentration time-profile of ganciclovir for 75 h at a dosing interval of 28.1 h for GCM and 12.3 h for ganciclovir solution

**Fig. 6:**
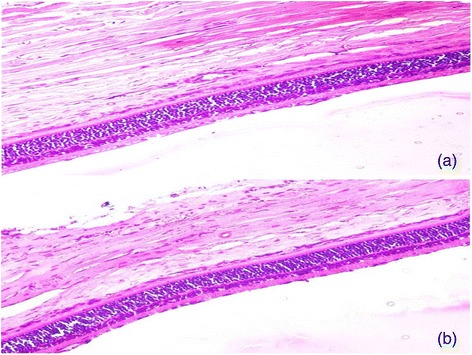
Photomicrographs of histological slides of rat retina (**a**) GCM and (**b**) ganciclovir solution

Using the best fit model parameters, the aqueous humor concentration-time profile of GCM and ganciclovir solution was simulated with every 28.1 and 12.8h, respectively (Fig. 5). The simulated concentration-time profile shows that in duration of 75 h, the ganciclovir solution require six ocular instillations compared to three instillations of the GCM formulation. Thus, GCM minimizes dosing frequency by sustained ganciclovir release for better efficacy. The photomicrograph of GCM and ganciclovir solution treated rat retina showed normal organization and cytoarchitecture (Fig. 6). The photomicrograph of GCM and ganciclovir solution showed inner layer of the retina, which was covered by nerve fibers followed by a layer of ganglion cells, an inner plexiform layer, inner nuclear layer, outer plexiform layer, outer nuclear layer, inner and outer segments of the rods, cones and sclera.

## Conclusions

The development of ganciclovir loaded chitosan microspheres was found to be ideal for ocular delivery. The microspheres showed Fickian type of drug release, and the XRD and stability studies showed favorable results. The GCM showed significant increase in AUC and C_max_ compared to ganciclovir solution. The C_max_/MIC_90_, AUC_0–24_/MIC_90_, AUC above MIC_90_ and T above MIC_90_ were higher in the GCM. Further, the *in vivo* ocular pharmacokinetic studies along with the histopathology report demonstrated the efficacy and tolerability of the formulation. Hence, the formulation significantly offered sustained drug release and improved intraocular bioavailability of ganciclovir in *Wistar* rats.
